# RNA and Toll-Like Receptor 7 License the Generation of Superior Secondary Plasma Cells at Multiple Levels in a B Cell Intrinsic Fashion

**DOI:** 10.3389/fimmu.2019.00736

**Published:** 2019-04-05

**Authors:** Caroline C. Krueger, Franziska Thoms, Elsbeth Keller, Fabiana M. S. Leoratti, Monique Vogel, Martin F. Bachmann

**Affiliations:** ^1^Department of BioMedical Research, University of Bern, Bern, Switzerland; ^2^Department of Immunology RIA, University Hospital Bern, Bern, Switzerland; ^3^Department of Dermatology, University Hospital Zurich, Schlieren, Switzerland; ^4^Nuffield Department of Medicine, The Henry Wellcome Building for Molecular Physiology, The Jenner Institute, University of Oxford, Oxford, United Kingdom

**Keywords:** memory B cells, secondary plasma cells, virus-like particles, toll-like receptor 7, anti-viral immunity, adaptive immunity

## Abstract

Secondary plasma cells (PCs) originate from memory B cells and produce increased levels of antibodies with higher affinity compared to PCs generated during primary responses. Here we demonstrate that virus-like particles (VLPs) only induce secondary PCs in the presence of toll-like receptor (TLR) 7 and if they are loaded with RNA. Furthermore, adoptive transfer experiments demonstrate that RNA and TLR7 signaling are required for secondary PC generation, both at the level of memory B cell as well as PC differentiation. TLR7-signaling occurred in a B cell intrinsic manner as TLR7-deficient B cells in an otherwise TLR7-competent environment failed to differentiate into secondary PCs. Therefore, RNA inside VLPs is essential for the generation of memory B cells, which are competent to differentiate to secondary PCs and for the differentiation of secondary PCs themselves. While we have not tested all other TLR or non-TLR adjuvants with our VLPs, these data have obvious implications for vaccine design, as RNA packaged into VLPs is a simple way to enhance induction of memory B cells capable of generating secondary PCs.

## Introduction

Antibodies are the critical effector molecules induced by prophylactic vaccination and are responsible for anti-viral and anti-bacterial protection. PCs are the principle cell type producing antibodies. A number of different antibody forming cells (AFCs) have been described. At an early stage of the primary immune response, short-lived AFCs derived from marginal zone or follicular B cells are found in extra-follicular foci in secondary lymphoid structures (1). A second wave of PCs is generated by the germinal center (GC) reaction of which some are also short-lived. However, a subset of GC derived PCs is long-lived and resides in secondary lymphoid organs as well as bone marrow (BM) for months and even years (2–4). It has been known for decades that memory B cells can differentiate to PCs after secondary antigen encounter (5, 6). We have recently described the particular phenotype of these PCs, which we coined secondary PCs, as they derive from memory B cells during secondary responses, in a VLP immunization model (7). In contrast to PCs induced during primary responses, they produce increased levels of high affinity antibodies. Unexpectedly, secondary PCs are short-lived and disappear a few days after their induction (Krueger et al., under review^1^).

The Th cell dependence of PC induction varies with the type of B cell progenitor. B1 cells, which provide only 1% of splenic B cells and are usually found in the peritoneal and pleural cavity, are a major source of natural antibodies produced in a Th cell independent manner (8). Early extra-follicular PCs, which often produce IgM, may be induced in the absence of T cell help in many cases, in particular if Th cell independent antigens are used for immunization (9). In contrast, GC-derived PCs are often isotype-switched and their generation requires T cell help and CD40L (10, 11). As opposed to GC-derived primary PCs, secondary PCs derived from memory B cells can be induced in the absence of CD40L (12). Hence, secondary PCs provide an early wave of antibodies in a relatively Th cell independent fashion during secondary responses, in a way similar to the short-lived extra-follicular PCs induced during primary responses.

Most antibody responses are driven by follicular Th cells (13, 14). However, presence of TLR-ligands, such as RNA, may overcome the requirement of follicular Th cells and other Th cells may take over (15–21).

There is a large number of adjuvants that are able to induce strong and long-lived B cell and antibody responses (22). Even though TLR-ligands are potent enhancers of B cell responses (23, 24), there is not an absolute requirement for the presence of TLR-ligands in order to induce protective B cell responses. Nevertheless, TLR-ligands play an important role for the generation of antibody responses during natural infections and many natural or artificial TLR-ligands are in development for adjuvants formulation (25–28) often in combination with classical adjuvants such as Alum (29). Monophosphoryl lipid A (MPL), a synthetic TLR4-ligand, is part of marketed vaccines since decades (30–32) and CpGs, a synthetic ligand for TLR9, have recently been approved for use in combination with hepatitis B vaccine (33). Furthermore, natural TLR-ligands are components of many widely used vaccines; in particular RNA, which is part of almost all live and inactivated viral and bacterial vaccines (34, 35). Single stranded RNA (ssRNA) is recognized by TLR7/8 in the endosome and RNA-sensing molecules in the cytosol and enhances antibody responses in many ways. B cells recognize RNA associated with the antigen via TLR7/8 and respond with increased production of IgG and in particular with a shift to the IgG2a subclass (17, 36, 37), enhanced B cell proliferation and increased BCR hypermutation (38, 39). This mechanism is dependent on TLR7-signaling in B cells and independent of RNA sensing in DCs (17). Similar B cell intrinsic pathways have been described for TLR9 (36, 37) which drives antibody responses in an IRF5-dependent way (40) and promotes B cell survival (28). The IgA subclass is particularly interesting with respect to TLR7-signaling, as systemic IgA responses need TLR signaling in B cells, while mucosal IgA responses need TLR signaling in DCs (41, 42). In addition, it has recently been shown that IgG responses against gram-negative bacteria require RNA-sensing in DCs followed by activation of TRIF and further downstream the inflammasome pathway (35, 43). The requirement for B cell intrinsic TLR signaling varies with time and is more important early than late during the GC response (34, 39), a finding that is consistent with the fact that the early GC response is more important than the late response to drive long-lived antibody responses (44). Furthermore, recent work indicated a temporal switch in GC reactions, where memory B cells are shown to emerge early during the response, whereas long lived PCs are a late output of the GC (20).

We have previously shown that immunization with VLPs derived from the RNA bacteriophage Qβ elicits strong and sustained IgG antibody responses with a prominent role for packaged *E.coli* RNA in driving class switch to IgG2a and IgA antibodies (42, 45–47). During recall responses, MBCs rapidly and quantitatively differentiate into secondary PCs (7). Here we show that RNA and TLR7-signaling in B cells synergize for the regulation of the secondary PC response. Absence of RNA or TLR7-signaling resulted in complete failure to generate memory B cells competent of forming secondary PCs. Moreover, stimulation of memory B cells generated in the presence of RNA, also failed to result in secondary PC induction in the absence of TLR7-signaling during recall. Hence, generation of secondary PCs is regulated by RNA and TLR7-signaling at multiple levels.

## Materials and Methods

### Study Design

**Figure 1:**
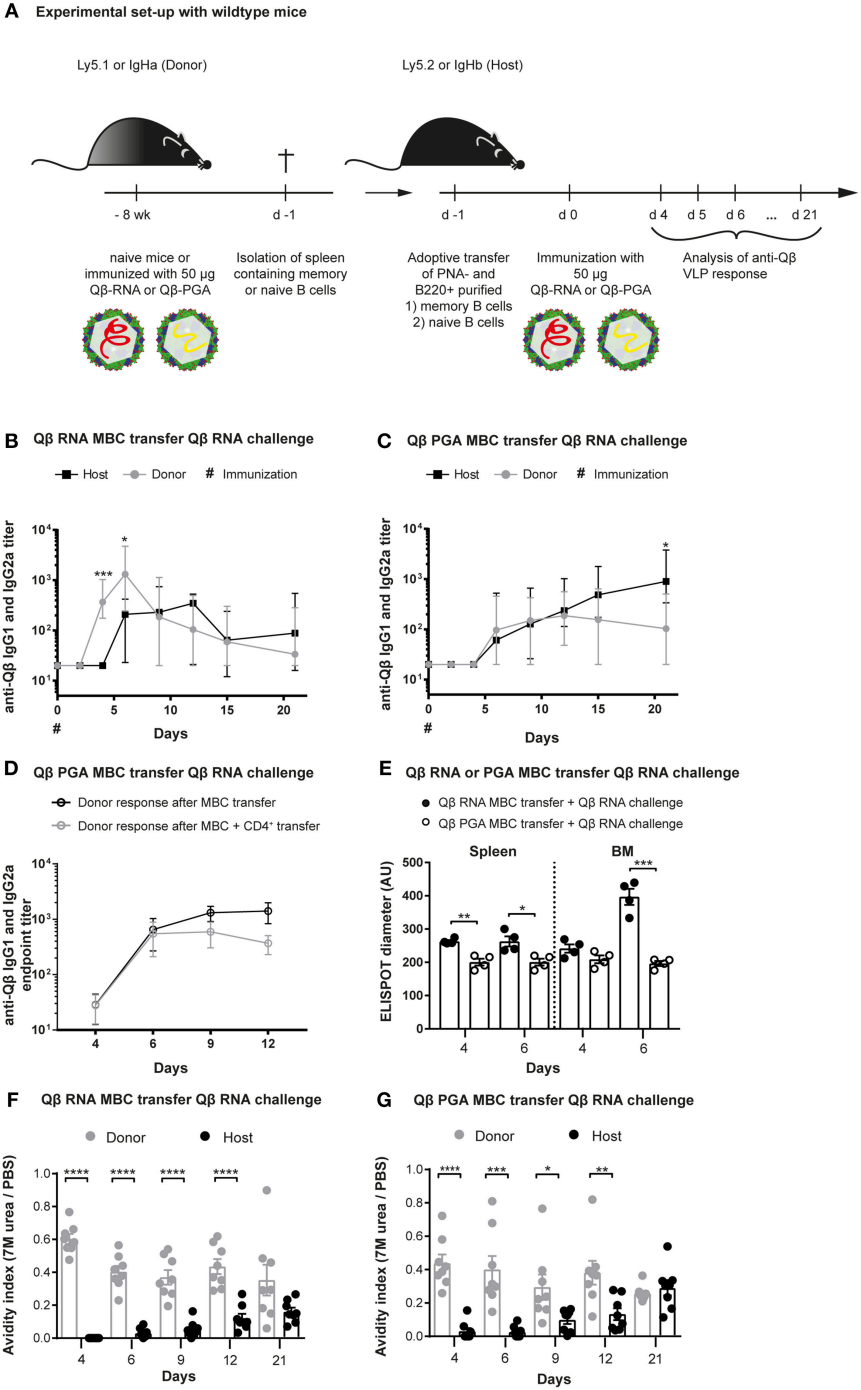
Memory B cells generated in presence of bacterial RNA generate secondary PCs after challenge with VLPs containing RNA. **(A)** Congenic mice (Ly5.1 or IgHa) were immunized with 50 μg Qβ VLPs containing RNA **(B,E,F)** or polyglutamic acid (PGA) **(C–E,G)** i.v. Eight weeks after immunization spleens of immunized and naïve mice were isolated and PNA^−^ B220^+^ **(B,C,E–G)** and CD4^+^ **(D)** MACS purified cells were transferred into host mice (Ly5.2 or IgHb). Recipient mice were immunized with 50 μg Qβ-RNA or Qβ-PGA i.v. 1 day after the transfer. Spleens, bone marrow, and serum were taken at several time points after challenge. **(B,C)** The anti-Qβ IgG1 and IgG2a antibody titers in the serum were determined by ELISA. Ha and Hb allotype specific detection antibodies were used to discriminate between donor (IgHa, gray circles) and host (IgHb, black squares) responses. **(D)** The endpoint titer of anti-Qβ IgG1 and IgG2a antibodies in the serum was determined by ELISA. Donor-derived responses after memory B cell (black open circles) or memory B cell and memory CD4^+^ T cell (gray open circles) transfer were detected using Ha allotype specific detection antibodies. **(E)** Quantification of the spot diameter in ELISPOT assays after transfer of memory B cells induced with 50 μg Qβ-RNA (black circles) or Qβ-PGA (open circles) and challenge with 50 μg Qβ-RNA. A modified ELISA was performed to determine the avidity index of the sera after transfer of memory B cells generated in presence **(F)** or absence **(G)** of bacterial RNA. Mean with SEM. *P*-values were obtained using an unpaired *t*-test. ^*^p < 0.05, ^**^p < 0.01, ^***^p < 0.001, ^****^p < 0.0001. *n* = 4 mice per group. Data representative of 2 independent experiments, except for D and E, where only one experiment was performed.

The goal of this study was to further characterize secondary PCs, which were generated by MBCs after Ag challenge. To achieve this, adoptive transfers in allotypic mice (Ly5.1/Ly5.2, IgHa/IgHb, TLR7 KO/WT, and TLR7 KO BM chimeras/WT BM chimeras) were performed. This enabled us to study primary and secondary immune responses in the same animal. All mice were kept according to cantonal veterinary guidelines at the central animal facility (Department of Biomedical Research) of the University of Bern and controlled laboratory experiments were performed in accordance with ethical principles and guidelines of the Cantonal Veterinary Office Bern, Switzerland. Animals were randomly assigned to the different groups. MBCs were generated by VLP immunization of mice. The control naïve mice remained untreated. At the same time, B cells were isolated from memory and naive mice and transferred into recipients. Upon immunization with VLPs, serum samples, spleens, and BM were collected and subjected to ELISA, ELISPOT, and FCM analysis. The investigators who performed the experiments, assessed, analyzed, and quantified the results were not blinded and aware of which group a sample was taken from. Individual groups consisted of four mice. All experiments were performed in at least two independent biological replicates. For the ELISA and ELISPOT in Figures 1D,E and day 6 FCM experiment only one replicate was performed. Data were collected at previously determined time points. All data were included in the analysis.

### Mice

C57BL/6JRccHsd wildtype mice were purchased from Envigo (Horst, The Netherlands). The IgHa [B6.Cg-Gpi1 <a> Thy1 <a> Igh <a> (Stock No. 001317)] mouse strain was purchased from the Jackson Laboratory (USA). We thank Prof. Annette Oxenius for the kind donation of the Ly5.1 (B6.SJL-Ptprc <a> Pepc <b>/BoyJ) mouse strain, Prof. Dr. Pål Johansen for the kind donation of the TLR7 KO (B6.129P2-Tlr7tm1Aki) mouse strain and Prof. Andrew Macpherson for the kind donation of the JH KO (B6.129P2-Igh-Jtm1Cgn/J) mouse strain.

### Generation of BM Chimeras

C57BL/6JRccHsd wildtype mice were lethally irradiated by the application of 1,300 cGy as a split dose of 2 × 650 cGy with a 4 h interval, using a Gammacell 40 (GC40) research irradiator (Best Theratronics). Irradiated mice were reconstituted with 25 × 10^7^ donor bone marrow cells, consisting of 80% JH KO and 20% TLR7 KO or 20% C57BL/6JRccHsd WT cells, respectively, i.v. Antibiotics [Baytril (1.25 ml/l) and Bactrim Nopil (5 ml/l)] were supplied in the drinking water for 2 weeks. After 6 weeks, reconstitution of the BM chimeras was analyzed by staining of B cell (B220) and T cell (CD3) markers using FCM. BM chimeras were immunized with 50 μg Qβ VLPs formulated in 150 μl phosphate buffer intravenously 10 weeks after irradiation.

### Antigen

The bacteriophage derived Qβ virus-like particles (VLPs) self-assemble and enclose bacterial RNA during their production in *E. coli*. The purification process is described elsewhere (48). VLPs without RNA were generated by disassembling the particles in presence of DTT in acidic conditions. This results in dimer formation, which were purified by size exclusion chromatography. Afterwards, the dimers were reassembled with polyglutamic acid (PGA) (17). VLPs containing B type CpGs (1668) were prepared as described previously (49, 50). Briefly, RNA inside the VLPs was digested using RNAse A (1.2 mg/ml for 3 mg/ml VLPs) for 3 h at 37°C. RNA digestion was confirmed using a 1% agarose gel stained with peqGreen dye. VLPs were repackaged by adding 1.125 μg CpG oligonucleotides to 20 μg RNAse digested VLPs for 3 h at 37 °C and repackaging was confirmed on a 1% agarose gel.

### Immunization

To induce primary immune responses and generate memory B cells against the VLPs, mice were immunized intravenously (i.v.) with 50 μg Qβ-RNA or Qβ-PGA. To challenge adoptively transferred MBC or naive cells, recipient mice were immunized with 50 μg of either Qβ-RNA, Qβ-PGA or Qβ-CpG i.v. For administration the VLPs were formulated in 150 μl phosphate buffer.

### Adoptive Transfer

MBCs were generated by immunization of congenic donor mice (Ly5.1, IgHa, TLR7 KO, WT, or BM chimeras). At least 8 weeks after immunization donor mice were sacrificed and spleens isolated in RPMI media containing 2% FCS and antibiotics. A single cell suspension of the spleens was prepared and red blood cells were lysed using ACK buffer (0.15 M ammonium chloride, 0.01 M potassium hydrogen carbonate, pH 7.2–7.4). The splenocytes were PNA^−^ and B220^+^ MACS purified. For PNA negative purification splenocytes were labeled using PNA-biotin (Vector Labs, B-1075) and PNA^+^ cells were depleted by Strepravidin MicroBeads (Milteny Biotec, 130-048-101) according to the manufacturer's protocol. Positive selection using B220 and CD4 MicroBeads (Milteny Biotec, 130-049-501, 130-117-043) was performed according to the manufacturer's protocol.

Purified cells from 1/3 of a donor spleen (Ly5.1, IgHa, TLR7 KO, WT, or BM chimeras ~1–3 × 10^6^ cells of which ~0.05–0.1% are specific for the antigen) were adoptively transferred i.v. into congenic host mice (Ly5.1, Ly5.2, or IgHb). Control mice received PNA^−^ and B220^+^ purified splenocytes from naïve congenic mice. One day after memory B cell transfer host mice were challenged with 50 μg of either Qβ-RNA, Qβ-PGA or Qβ-CpG i.v. formulated in 150 μl phosphate buffer.

### ELISPOT

Spleens from mice after adoptive transfer were isolated and a single cell suspension was prepared. To collect BM cells, tibia and femur were flushed with RPMI media containing 2% FCS and antibiotics. After red blood cell lysis with ACK buffer, cell numbers of splenocytes and BM cells were determined using the Cellometer mini (Nexcelom, USA). 5 × 10^5^ cells were seeded per well on MAIPS ELISPOT plates (Millipore, MAIPS4510) previously coated with 10 μg/ml Qβ VLPs overnight at 4°C and blocked with 2% BSA in PBS for at least 2 h. After performing a 2-fold dilution series, cells were incubated for 5 h at 37°C and 5% CO_2_. Subsequently cells were washed off and bound specific antibodies produced by PCs were detected using a goat anti-mouse IgG antibody (EY laboratories, AT-2306-2) followed by a donkey anti-goat alkaline phosphatase secondary antibody (Jackson Immunoresearch, 705-055-147). Spots were visualized by the AP Conjugate Substrate Kit (BioRad, 1706432) and counted using an EliSpot Reader (AID, Germany).

### ELISA

Serum samples were obtained from blood collected at the indicated time points during experiments using Microtainer tubes (BD, 365967). Corning half area 96 well plates were coated with 1 μg/ml Qβ VLPs overnight at 4°C. Sera were 1:10 pre-diluted and 1:4 further serial diluted to analyse a total of 7 dilutions per sample. Qβ-specific antibodies were detected using mouse anti-mouse IgG for both allotypes. IgHa-specific (biotin ms anti-ms IgG1[a] (10.9), biotin ms anti-ms IgG2a[a] (8.3) from BD) and IgHb-specific (biotin ms anti-ms IgG1[b] (B68-2), biotin ms anti-ms IgG2a[b] (5.7) from BD) antibodies were detected using horseradish peroxidase (HRP) labeled streptavidin (Jackson ImmunoResearch, 016-030-084).

Total Qβ-specific antibodies were detected using goat anti-mouse IgG-HRP (Jackson ImmunoResearch, 115-035-071).

The absorbance readings of the tetramethylbenzidine (TMB) color reaction at 450 nm for the serum samples were interpreted as OD50 or endpoint antibody titers. The OD50 antibody titers are defined as the reciprocal of the dilution that reaches half of the maximal optical density (ODmax). The endpoint antibody titers are defined as the reciprocal of the last dilution above the threshold, which is set above the background level.

### Avidity ELISA

Serum samples were obtained from blood collected at the indicated time points during experiments using Microtainer tubes (BD, 365967). Corning half area 96 well plates were coated with 1 μg/ml Qβ VLPs overnight at 4°C. Sera of the different time points were applied with a 1:10 pre-dilution and 1:4 further serial diluted. After 1 h incubation, the sera were washed off and the plates washed 3 times 5 min either with 7 M urea in PBST (PBS containing 0.05% Tween20) or PBST only. Qβ specific antibodies were detected using mouse anti-mouse IgG for both allotypes. IgHa-specific (biotin ms anti-ms IgG1[a] (10.9), biotin ms anti-ms IgG2a[a] (8.3) from BD) and IgHb-specific (biotin ms anti-ms IgG1[b] (B68-2), biotin ms anti-ms IgG2a[b] (5.7) from BD) antibodies were detected using horseradish peroxidase (HRP) labeled streptavidin (Jackson ImmunoResearch, 016-030-084). Total Qβ-specific antibodies were detected using goat anti-mouse IgG-HRP (Jackson ImmunoResearch, 115-035-071). The absorbance readings of the tetramethylbenzidine (TMB) color reaction at 450 nm served as basis for avidity index calculation. The avidity index (AI) was calculated by AI_x_ = OD (dilution x) + urea/OD (dilution x)–urea.

### Flow Cytometry (FCM)

For FCM staining spleens of mice after adoptive transfer were isolated in RPMI supplemented with 2% FCS and antibiotics and a single cell suspension was prepared. Red blood cells were lysed using ACK buffer prior to staining. Fc receptors were blocked using an anti-CD16/32 antibody (2.4G2, BD). To discriminate Qβ-specific plasma cells (PCs) from Qβ-specific activated and CS B cells, surface immunoglobulins (Ig) of specific cells were blocked using unlabelled Qβ VLPs. PCs were further stained with and characterized as IgM (polyclonal, Jackson ImmunoResearch), IgD (11-26c (11-26), eBioscience), CD4 (H129.19, BD), CD8 (53-6.7, BD), GR1 (RB6-8C5, BD), CD11b (M1/70, BD), CD11c (HL3, BD) negative (all antibodies labeled with PE), and B220-PE-Cy7 (RA3-6B2, BD) low. To detect Qβ specific PCs by intracellular staining of specific Ig, splenocytes were permeabilized using FACS lysing solution (BD, 349202) containing 0.04% Tween20 and stained with Alexa Flour 488 labeled Qβ VLPs. The congenic marker Ly5.1 (antibody labeled with APC, A20, eBioscience) identified all transfer derived B cells.

Qβ VLPs were labeled with the Alexa Flour 488 protein labeling kit (Thermo Fisher Scientific, A10235) according to the manufacturer's instructions.

### Statistics

Statistical analysis was performed using GraphPad Prism Version 7.01 (GraphPad Software, USA). Statistically significant differences between two groups were calculated using unpaired *t*-tests. Statistical significance was defined as *p* < 0.05.

## Results

### RNA Drives the Generation of Memory B Cells Competent of Forming Secondary PCs

We have previously demonstrated that vaccination with Qβ VLPs containing bacterial RNA leads to the formation of long-lasting humoral memory. Upon immunization, isotype-switched memory B cells as well as PCs are generated in a Th cell-dependent manner (7, 12, 44, 45). During secondary responses, VLP specific memory B cells do not re-enter GCs but differentiate to short-lived secondary PCs independent of T cell help (7, 12). The hallmark of secondary PCs is increased production of high affinity antibodies early after activation (Krueger et al., under review). To further study the mechanism of secondary PC generation, adoptive transfers of memory B cells using congenic mice were performed. Briefly, memory B cells were generated by immunizing wildtype (WT) donor mice with Qβ VLPs containing either bacterial RNA (Qβ-RNA) or polyglutamic acid (Qβ-PGA), a negatively charged polymer serving as surrogate for RNA to enable VLP-assembly, which, however, does not stimulate TLRs (Figure 1A). Purified memory B cells of immunized or naïve donor mice were transferred into congenic recipient mice expressing a different IgH- or Ly5- allotype. We did not co-transfer memory CD4^+^ T cells after Qβ-RNA priming, as we have previously observed that their presence has no influence on VLP specific memory B cell responses (7, 12). Upon cell transfer and challenge with Qβ-RNA the specific VLP antibody response of transferred memory B cells and host B cells was assessed within recipient mice (Figures 1B,C). As observed before, when memory B cells were induced with Qβ-RNA and challenged with Qβ-RNA, the on- and offset as well as the magnitude of the antibody response derived from memory B cells was significantly faster and higher compared to the host's primary antibody response (Figure 1B). Memory B cell derived IgG titers raised within 4 days and peaked early at day 6 post-immunization. In contrast, the host's primary response became detectable on day 6 and peaked at day 12 after immunization. However, if memory B cells were generated with Qβ-PGA instead of Qβ containing RNA, memory B cell derived antibody responses were not increased but rather similar to the host‘s antibody response after challenge with Qβ-RNA (Figure 1C; Figure S1A). Therefore, the memory antibody response resembled the one of the primary response if VLPs deprived of RNA were used for memory B cell generation indicating that RNA inside the VLPs was crucial for enhanced antibody responses during secondary Ag challenges. A similar donor response was seen when Qβ-PGA memory B cells were transferred in presence or absence of memory CD4^+^ T cells (Figure 1D) and challenged with Qβ-RNA. Consequently, like in Qβ-RNA secondary responses, co-transfer of memory CD4^+^ T cells had no influence on the VLP specific memory B cell response. Of note, memory B cells generated with VLPs containing RNA also failed to generate increased IgG levels if the boost was performed with Qβ devoid of RNA (Figure S2A). Thus, enhanced IgG responses were only detectable when memory B cells were generated and boosted with Qβ-RNA (Figures 1B,C; Figures S1A, S2A-C). The increased IgG response produced by memory B cells generated in presence of RNA could also be verified in ELISPOT assays of spleen and BM, where the spot diameter correlates with the amount of antibodies produced by one PC (Figure 1E).

We further investigated the antibody response by performing avidity ELISAs (Figures 1F,G). The avidity index was determined by a modified ELISA where washes with 7 M urea were performed, which dissociates low avidity antibodies but high avidity antibodies remain bound. The avidity index of secondary response antibodies after Qβ-RNA induced memory B cell transfer and Qβ-RNA re-stimulation was high as of day 4 and stayed significantly higher compared to the primary response antibodies until day 21 (Figure 1F). A similar, but less pronounced, observation of the avidity increase was made, when Qβ-PGA induced memory B cells were transferred and re-stimulated with Qβ-RNA (Figure 1G). In strong contrast, primary response antibodies reach comparable avidity only after 21 days in the absence of memory B cell transfer (Figure S1B). These data indicate that RNA is required to generate memory B cells capable of generating secondary PCs but affinity maturation occurred to a large degree in the absence of RNA.

### Presence of Bacterial RNA During Challenge of Memory B Cells Is Essential to Generate Secondary PCs

**Figure 2:**
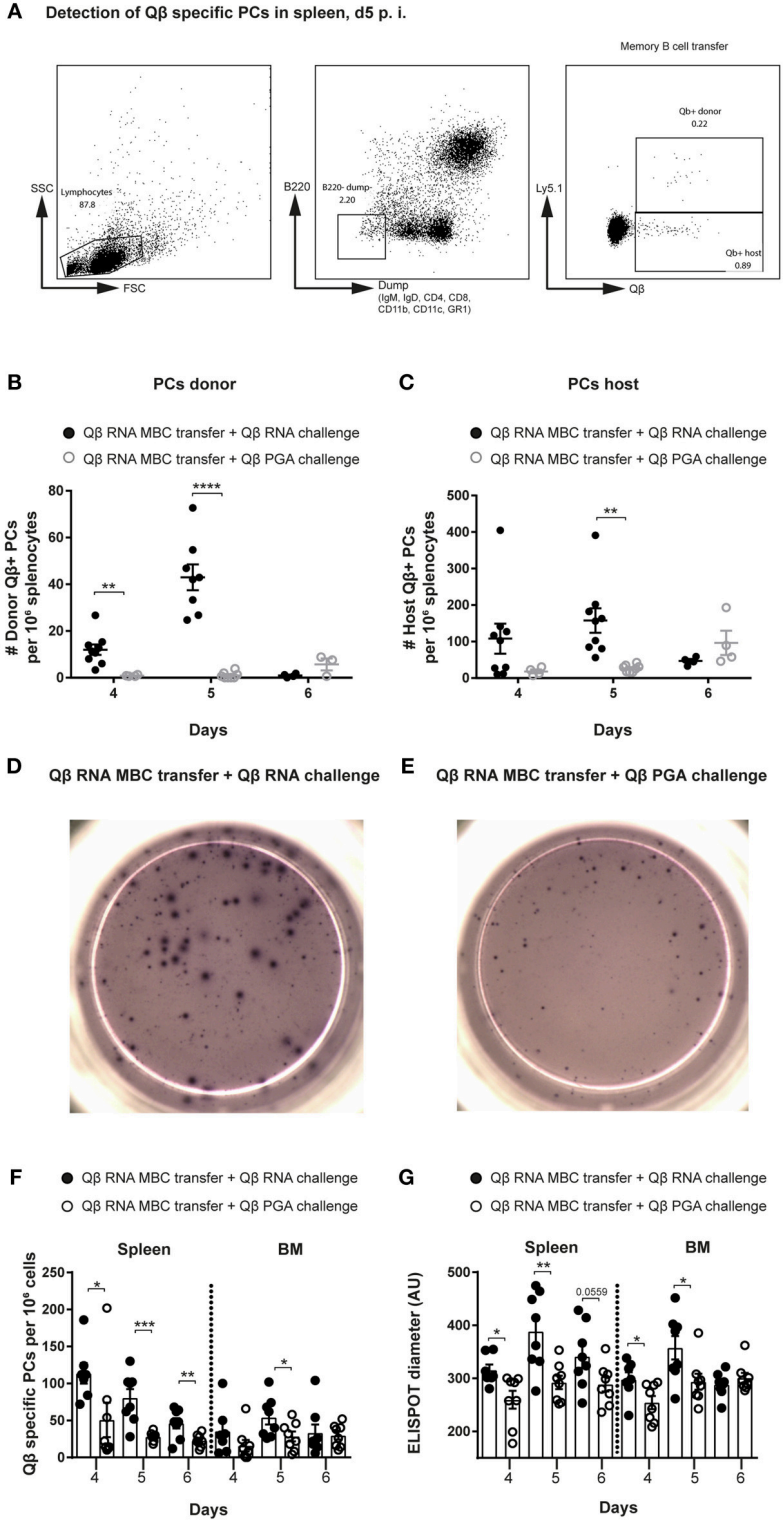
Presence of bacterial RNA during challenge of memory B cells is essential to generate secondary PCs. Memory B cells were generated by immunizing Ly5.1 mice (donor) with 50 μg Qβ-RNA. One day after transfer of MACS purified memory B cells, allotypic Ly5.2 hosts were challenged with 50 μg Qβ-RNA or 50 μg Qβ-PGA, respectively. **(A)** Representative FCM plots for the gating strategy to identify Qβ-specific PCs in the spleen 5 days after transfer and challenge. B220^low^ cells not expressing IgM, IgD, CD4, CD8, CD11b, CD11c, or GR1 were analyzed for their intracellular binding of labeled Qβ VLPs. The congenic Ly5 marker was used to discriminate transfer from host derived PCs. **(B,C)** FCM analysis of the specific PC compartment at day 4, 5, and 6 after transfer of memory B cells induced with Qβ-RNA and challenged with Qβ-RNA (black circles) or Qβ-PGA (gray circles). Number of Qβ-specific donor **(B)** or host **(C)** derived PCs within the B220^low^, IgM, IgD, CD4, CD8, CD11b, CD11c, and GR1 negative compartment, binding Qβ intracellularly after membrane permeabilisation. Representative images of ELISPOTs of the spleen at day 5 after Qβ-RNA memory B cell transfer and Qβ-RNA **(D)** or Qβ-PGA **(E)** challenge. **(F)** Numbers of Qβ-specific PCs in spleen and BM 4, 5, and 6 days after memory B cell transfer and challenge with either Qβ-RNA (black circles) or Qβ-PGA (open circles) were determined by ELISPOT. **(G)** Quantification of the spot diameter in ELISPOT assays after memory B cell transfer and challenge with either Qβ-RNA (black circles) or Qβ-PGA (open circles). Mean with SEM. *P*-values were obtained using an unpaired *t*-test. ^*^p < 0.05, ^**^p < 0.01, ^***^p < 0.001, ^****^p < 0.0001. *n* = 4 mice per group. Data representative of 2 independent experiments, except for day 6 of B and C, where only one experiment was performed.

After induction of memory B cells with Qβ-RNA in Ly5 allotypic wildtype mice, MACS purified memory B cells were adoptively transferred into allotypic recipients, which were challenged with Qβ-RNA or Qβ-PGA 1 day after transfer (Figure 1A). Antigen-specific PCs were identified as B220^low^, IgM, IgD, CD4, CD8, CD11b, CD11c, GR1 negative and by intracellular Qβ binding after membrane permeabilisation using flow cytometry (FCM). Donor derived cells (Ly5.1^+^) were discriminated from host derived cells (Ly5.2^+^) using the Ly5 marker (Figure 2A). Donor, hence memory B cell derived Qβ-specific PCs, were significantly increased on day 4 and 5 after challenge with Qβ-RNA compared to Qβ-PGA (Figure 2B). This difference was less prominent looking at host derived antigen specific PCs generated early during the primary response (Figure 2C).

The increased PC response generated by transferred memory B cells boosted with Qβ-RNA in comparison to Qβ-PGA correlated with PC numbers detectable in spleen and BM analyzed by ELISPOT (Figures 2D–F). In addition, secondary PCs generated in presence of Qβ-RNA were capable to produce more antibodies shown by the increased spot diameter observed in ELISPOT analysis (Figures 2D,E,G). The peak of the PC number and spot diameter in spleen and BM after transfer of Qβ-RNA-primed memory B cells followed by challenge with Qβ-RNA was around day 4 and 5, thereafter PC numbers rapidly declined, demonstrating the short-lived nature of the secondary PCs. In contrast, boosting with Qβ-PGA resulted in slower but more sustained responses (Figure 2B, day 6). The early PC population detectable in spleen and BM after naïve cell transfer in control experiments generating a primary response was smaller and produced less antibodies (Figures S1C,D).

Thus, bacterial RNA as a TLR7 ligand is not only important in the generation of memory B cells but also during their differentiation to secondary PCs with increased ability to secrete high-affinity antibodies.

### RNA Induced TLR7 Signaling Must Be Present for Induction of Memory B Cells Competent to Differentiate Into Secondary PCs

**Figure 3:**
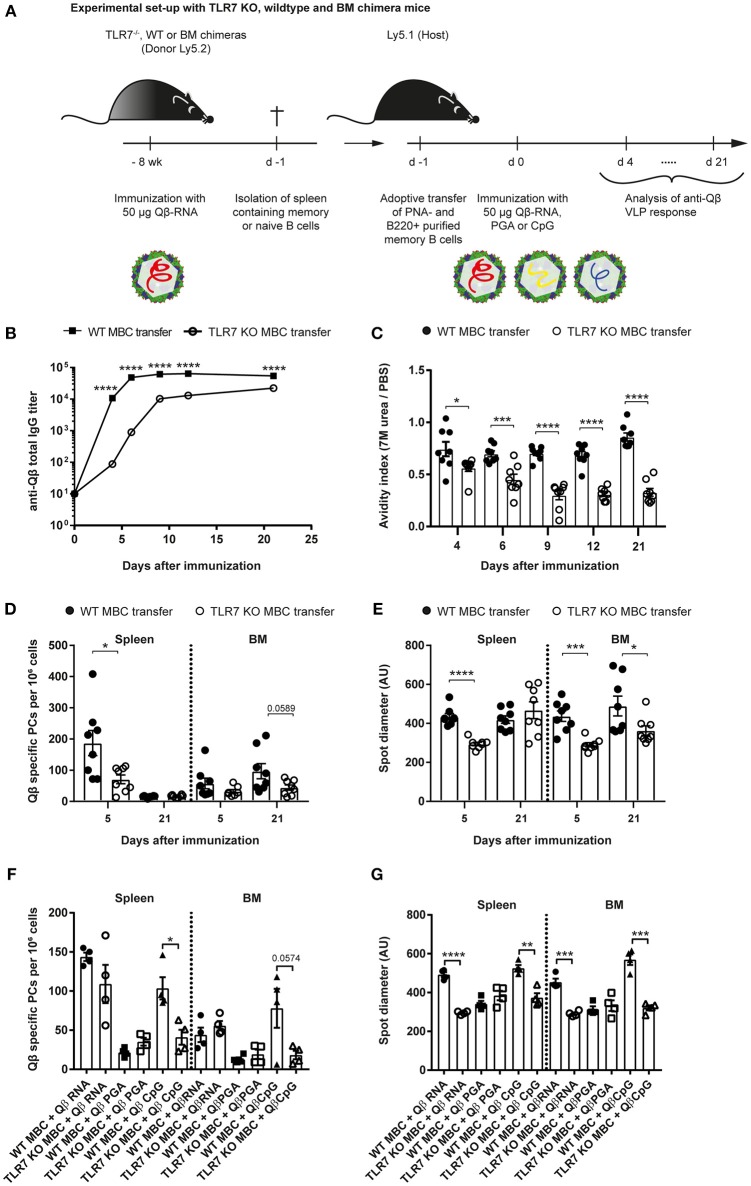
TLR7 must be present during memory B cell induction to generate secondary PCs after challenge with VLPs. **(A)** Memory B cells were induced in TLR7 KO, WT, or BM chimeras by immunization with 50 μg Qβ-RNA i.v. Eight weeks after immunization spleens were isolated and PNA^−^ B220^+^ MACS purified cells were transferred into host mice (Ly5.1). One day after the transfer, recipient mice were challenged with 50 μg Qβ-RNA **(B–E)** or Qβ-RNA, Qβ-PGA, and Qβ-CpG **(F,G)** i.v. Spleens, bone marrow, and serum were taken at several time points after challenge. **(B)** Qβ-specific total IgG titers after WT (black squares) or TLR7 KO (open circles) memory B cell transfer were determined by ELISA. **(C)** The avidity index after WT (black circles) or TLR7 KO (open circles) memory B cell transfer was determined by a modified ELISA. **(D,E)** ELISPOT assays were used to determine the number **(D)** of Qβ specific PCs and the spot diameter **(E)** produced by these in spleen and BM on days 5 and 21 after WT (black circles) or TLR7 KO (open circles) memory B cell transfer. **(F,G)** ELISPOT assays were used to determine the number **(F)** of Qβ specific PCs and the spot diameter **(G)** in the spleen and BM on day 5 after WT (black shapes) or TLR7 KO (open shapes) memory B cell transfer and challenge with Qβ-RNA, Qβ-PGA, or Qβ-CpG, respectively. Mean with SEM. *P*-values were obtained using an unpaired *t*-test. ^*^p < 0.05, ^**^p < 0.01, ^***^p < 0.001, ^****^p < 0.0001. *n* = 4 mice per group. Data representative of 2 **(B–E)** or 1 **(F,G)** independent experiments.

To assess whether TLR7 was involved in the generation of memory B cells competent to differentiate into secondary PCs, adoptive transfer experiments using TLR7 KO mice were performed. Memory B cells were induced in TLR7 KO or WT mice by Qβ-RNA vaccination. MACS purified memory B cells were then transferred into Ly5-allotypic WT mice and challenged with Qβ-RNA, Qβ-PGA, or Qβ-CpG (Figure 3A). As observed with Qβ-PGA (Figures 1C,G), the total anti-Qβ IgG titer and avidity was significantly lower at early time-points when TLR7 deficient memory B cells were transferred (Figures 3B,C). Therefore, the humoral immune responses observed in the absence of TLR7 correlated well with the data observed in the absence of RNA, indicating that memory B cells competent to differentiate to secondary PCs fail to differentiate in the absence of TLR7 signaling.

PC numbers and spot sizes in spleen and BM obtained from ELISPOT assays were consistent with the antibody responses and corroborated the findings using RNA-free VLPs (Figures 3D,E). Host mice that received WT memory B cells exhibited significantly increased PC frequencies in the spleen 5 days after VLP challenge (Figure 3D). This difference was less prominent in the BM. The spot diameter was significantly increased in spleen and BM after WT memory B cell transfer compared to TLR7 KO memory B cell transfer early during the response. This difference was maintained in the BM but gone in the spleen by day 21, likely because most secondary PCs had died by then (Figure 3E). In conclusion, as increased antibody production and spot size are a hallmark of secondary PCs, these cells were only generated when WT memory B cells were transferred, which could receive RNA-mediated TLR7 signals during their generation and re-stimulation.

To assess whether TLR7 KO memory B cells were competent to differentiate to secondary PCs, adoptive transfers of WT and TLR7 KO memory B cells and challenge with Qβ-RNA, Qβ-PGA, or Qβ-CpG were performed. Qβ-CpG contained B-type CpG oligodeoxynucleotides, which trigger myeloid differentiation primary response 88 (MyD88) signaling in B cells via TLR9. The memory B cell response toward the three challenge antigens was determined by ELISPOT at day 5 after challenge. TLR7 KO memory B cells exhibited a reduced capacity to generate secondary PCs in response to Qβ-RNA and Qβ-CpG challenge, shown by decreased spot number (Figure 3F) and spot size (Figure 3G) in the spleen and BM. The spot number and spot sizes generated were comparable to the ones of WT and TLR7 KO memory B cells challenged in the absence of any TLR ligand (Qβ-PGA) (Figures 3F,G), which failed to differentiate to secondary PCs (Figure 2). WT memory B cells on the contrary differentiated to secondary PCs after reactivation with Qβ-RNA and Qβ-CpG (Figures 3F,G), indicating that TLR9 stimulation can compensate for TLR7 when memory B cells were induced in presence of TLR7 ligands. Consequently, TLR7 signaling is indispensable during memory B cell priming for imprinting the ability of secondary PC generation after antigen challenge, as MyD88 signaling induced by TLR9 stimulation is not able to compensate for the defect seen after TLR7 KO memory B cell transfer. Moreover, presence of TLR7 or TLR9 signaling is sufficient for secondary PC formation after challenge of memory B cells generated in the presence of TLR7 signaling.

### B Cell Intrinsic TLR7 Signaling Is Needed to Generate Memory B Cells Capable of Differentiating to Secondary PCs

**Figure 4:**
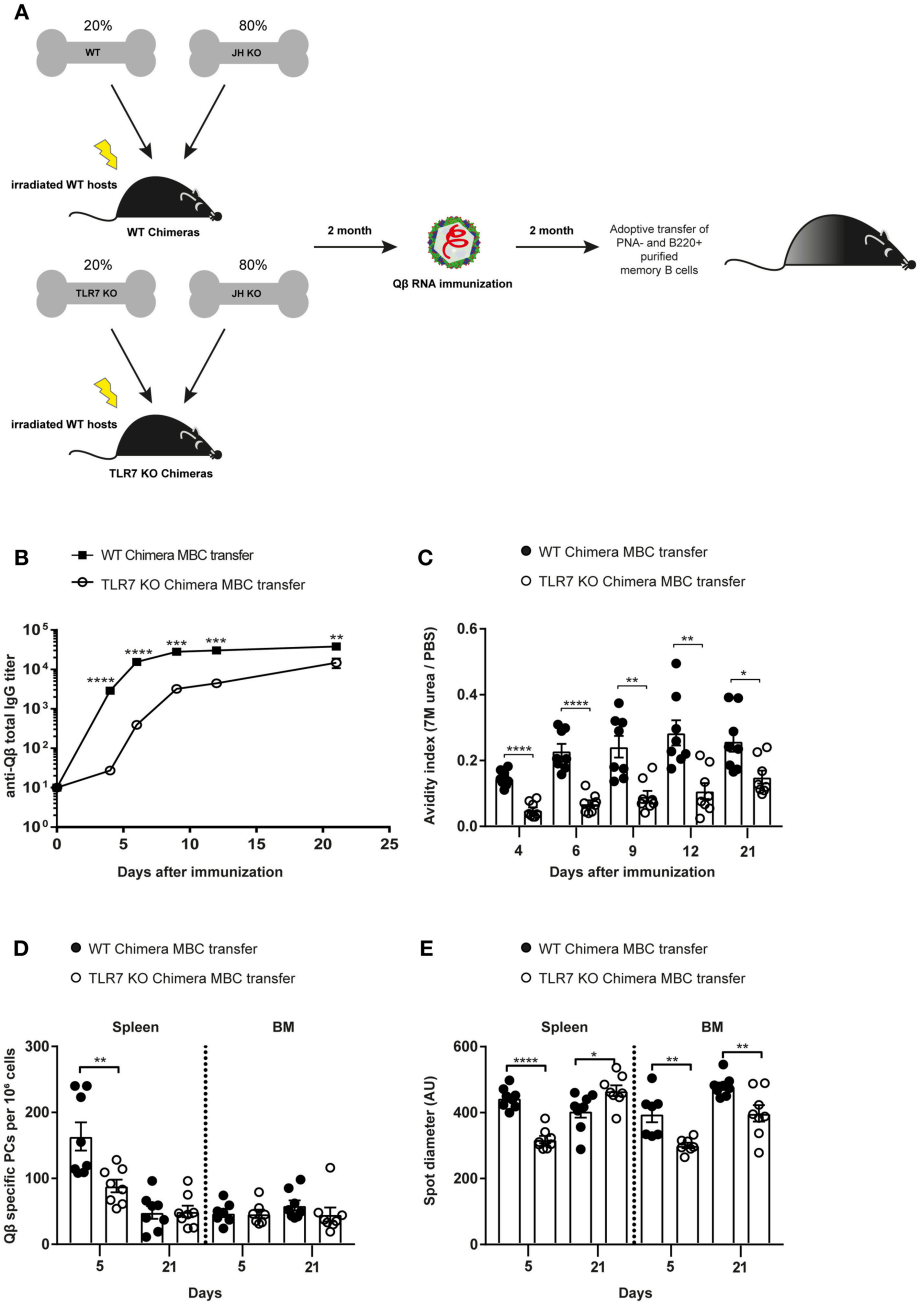
B cell intrinsic TLR7 signaling is needed to generate memory B cells capable of producing secondary PCs. **(A)** Mixed BM chimeric mice with 20% WT or TLR7 KO and 80% JH KO BM were generated. Eight weeks after reconstitution the WT and TLR7 KO chimeras were immunized with 50 μg Qβ-RNA. Memory B cells from BM chimeric mice were transferred into congenic Ly5.1 recipients after 8 weeks. Recipient mice were challenged with 50 μg Qβ-RNA 24 h after the adoptive transfer. **(B)** The anti-Qβ total IgG titer in the serum after WT chimera (black squares) or TLR7 KO chimera (open circles) memory B cell transfer was determined by ELISA. **(C)** The avidity index of antibodies from sera generated after WT chimera (black circles) or TLR7 KO chimera (open circles) memory B cell transfer and challenge was calculated after performing a modified ELISA. ELSIPOT assays of splenocytes and BM cells were performed to determine PC number **(D)** and spot diameter **(E)** 5 and 21 days after WT chimera (black circles) or TLR7 KO chimera (open circles) transfer and challenge. Mean with SEM. *P*-values were obtained using an unpaired *t*-test. ^*^p < 0.05, ^**^p < 0.01, ^***^p < 0.001, ^****^p < 0.0001. *n* = 4 mice per group. Data representative of two independent experiments.

To test whether TLR7 signaling was intrinsically required in B cells for secondary PC generation, mixed BM chimeras of JH knockout (KO) with WT or TLR7 KO BM were generated. TLR7 KO BM chimeras exclusively lack TLR7 in B cells, whereas WT BM chimera B cells were sufficient for TLR7 in all cells. Two months after reconstitution, both BM chimeras were immunized with Qβ-RNA (Figure 4A). MACS purified memory B cells were transferred into recipient WT mice and challenged with Qβ-RNA (Figures 3A, 4A). From day 4 after challenge the anti-Qβ total IgG titer as well as the affinity was significantly lower when memory B cells from TLR7 deficient BM chimeras were transferred (Figures 4B,C). The difference was particularly pronounced before day 9 after challenge, representing the time span when secondary PCs were dominating the response, providing the early wave of antibodies. As observed before, the differences became smaller at later time-points, as the host response developed (Figures 4B,C).

PC numbers and spot sizes examined in spleen and BM correlated well with the antibody responses (Figures 4B–E). After transfer of TLR7 KO BM chimera memory B cells, PC numbers in the spleen were significantly lower compared to WT BM chimera memory B cell transfer at day 5 after challenge. This difference was absent 21 days after challenge, again indicating that secondary PCs are short-lived (Figure 4D). Moreover, spot diameters, which correlate with the amount of antibodies produced by PCs, were smaller in spleen and BM at day 5 after challenge, when TLR7-deficient BM chimera memory B cells were transferred compared to WT BM chimera memory B cells (Figure 4E). Taken together, the data presented here clearly demonstrate that RNA and TLR7 signaling are required in a B cell intrinsic fashion for the generation of memory B cells and their differentiation to secondary PCs, which are capable to produce vast amounts of high affinity antibodies early during secondary responses.

## Discussion

B cell responses are controlled and regulated at multiple levels. As a key step, B cell specificity is cross-checked by available cognate T cell help and the presence of innate stimuli indicative of an infection. This is exemplified by the primary B cell response against viruses where specificity alone is driving the initial response by efficient cross-linking of BCRs by the repetitive viral surface (51). This results in a Th cell independent IgM response, which is, however, short-lived. Only the presence of cognate T cell help results in a GC response and isotype-switching. In this way, the immune system validates the BCR-signals by the presence of Th cells specific for the same antigen, which indicates that the recognized antigen is non-self. Presence of PAMPs, in particular TLR-ligands, is a second checkpoint, which implies that the antigen is not only non-self but most likely an infectious agent. This results in augmented antibody and Th cell responses. Here we demonstrate that TLR-signals, in particular TLR7, are also key in secondary B cell responses as they cause the differentiation of a subset of memory B cells capable to rapidly differentiate into secondary PCs upon re-exposure to the same antigen plus TLR7-ligand.

The adaptive immune system is confronted with the choice between speed and specificity. As clonal selection and, in the case of B cells, hypermutation need time to develope, high specificity comes at the cost of time. As many pathogens may be fatal within a week, highly specific antibody responses would be too late to provide protection (52). Broadly cross-reactive antibody responses, on the other hand, always carry the risk of non-desired recognition of self-antigens. The solution the immune system found during primary responses is the early and rapid generation of poly-reactive IgM antibodies, which are especially potent at recognizing repetitive surfaces due to their deca-valence. To balance the potential of IgM antibodies to cross-react, these responses are, however, short-lived and eventually replaced by highly specific bivalent IgG antibodies, which are controlled by cognate T cell help and presence of TLR-ligands.

We demonstrate here that secondary antibody responses may follow a similar pattern. Pre-existing, TLR7- and Th cell experienced memory B cells differentiate rapidly to secondary PCs, which produce large amounts of high affinity IgG antibodies. This differentiation occurs without direct interaction with cognate Th cells (12) but needs again the presence of RNA or CpG oligodeoxynucleotides to engage TLR7/9 signaling to ensure presence of an infectious agent. However, since the pathogen may have evolved over time, these high affinity IgG antibodies may primarily recognize the original pathogen rather than the current version. For this reason, similar as the IgM antibodies generated during the primary response, this early wave of secondary IgG antibodies is replaced by a more specific second wave of antibodies derived from naïve B cells, which again will require presence of Th and TLR7-ligands for optimal specificity.

The present data further underscore the importance of RNA-sensing in B cells. We and others have previously shown that TLR signaling in B cells drives primary B cell responses and is, at least for viral particles, more important than TLR7 signaling in DCs (16–19). Here we extend these findings to secondary B cell responses and demonstrate that B cell-intrinsic TLR7-signaling is essential for imprinting the ability to differentiate to secondary PCs, as vaccination with VLPs deprived of RNA induces affinity matured memory B cells which lack the potential to generate secondary PCs. Moreover, this signaling pathway is also key for driving the differentiation of these secondary PCs from memory B cells. Hence, TLR7 signaling in B cells is essential for the shaping of both primary and secondary B cell responses. These data have obvious implications for vaccine design as the major vaccines based on virus-like particles, those against hepatitis B virus, human papilloma virus and malaria do not package RNA. Future vaccine platforms may therefore be based on VLPs incorporating RNA in order to allow formation of memory B cells capable of differentiating into secondary PCs to provide the first wave of rapidly produced protective antibodies during re-infection.

## Author Contributions

CK, EK, and FL performed all experiments. CK, FT, and MB designed all the experiments. MV interpreted results and contributed to the scientific discussion. CK, FT, and MB wrote the manuscript. All authors read and commented on the manuscript.

### Conflict of Interest Statement

The authors declare that the research was conducted in the absence of any commercial or financial relationships that could be construed as a potential conflict of interest.

## Footnotes

^1^ Krueger CC, Thoms F, Keller E, Vogel M, Bachmann MF. Virus-specific secondary plasma cells produce elevated levels of high-affinity antibodies but are short lived. *Front Immunol.* (under review).
