# Supplementary material for: RNA and Toll-Like Receptor 7 License the Generation of Superior Secondary Plasma Cells at Multiple Levels in a B Cell Intrinsic Fashion
Source: Front Immunol. 2019 Apr 5;10:736. doi: 10.3389/fimmu.2019.00736 (PMC6467167; doi:10.3389/fimmu.2019.00736)
Supplement: Supplementary file 1 [file Data_Sheet_1.PDF]

Supplementary Materials

Figure S1

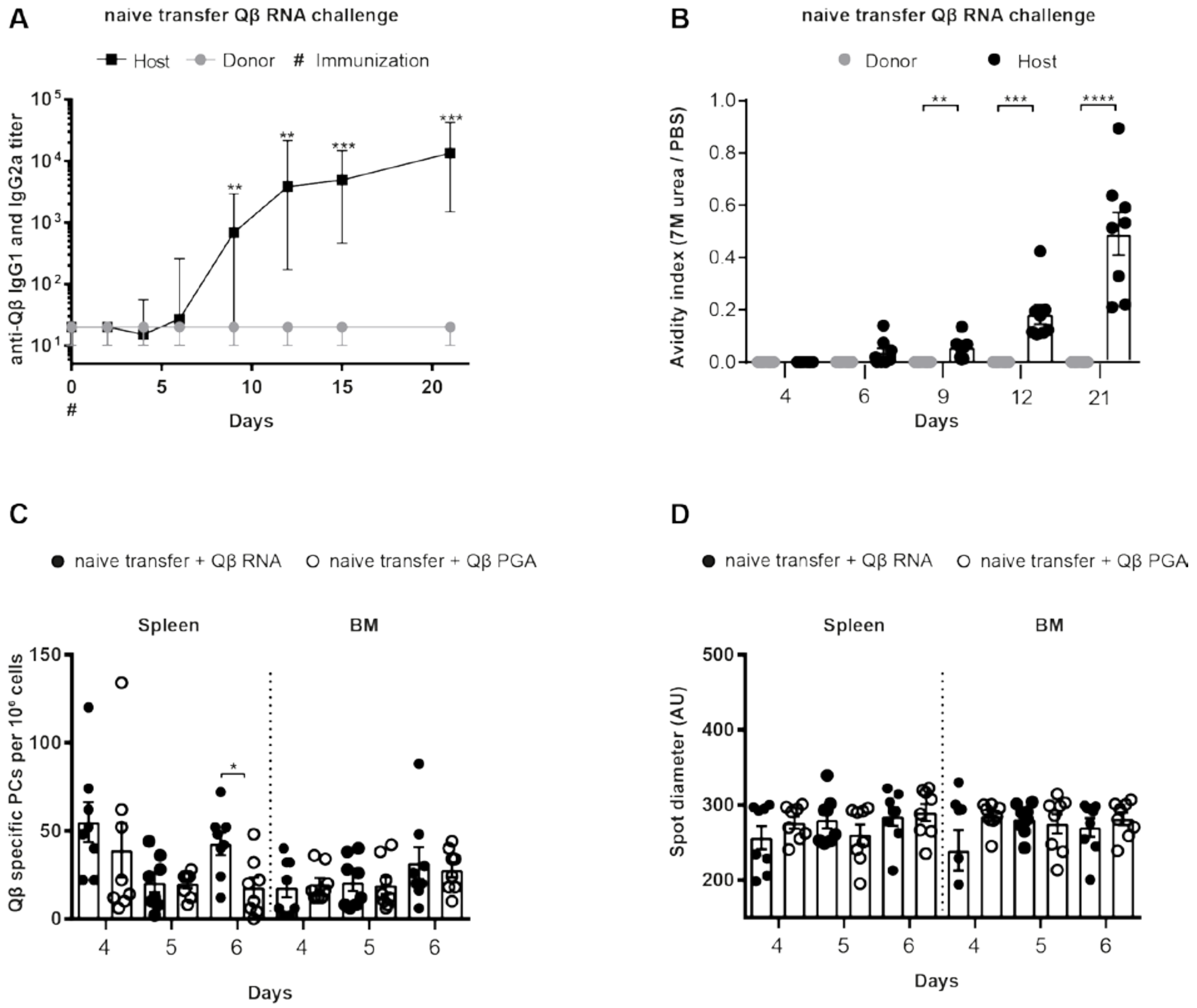

Figure S1. PC generation after naïve B cell transfer and Qβ-RNA challenge. Naïve B cells from IgHa (donor) mice were transferred into IgHb hosts, which were challenged with 50 µg Qβ-RNA one day after the transfer. (**A**) Anti-Qβ IgG1 and IgG2a antibody titers were determined in the sera by ELISA. (**B**) The avidity index of donor (IgHa) or host (IgHb) derived antibodies was determined by a modified ELISA. Using Ha and Hb allotype specific detection antibodies, donor and host derived antibodies could be discriminated. To quantify PC differentiation after naïve transfer and challenge with 50 µg Qβ-RNA or 50 µg Qβ-PGA, ELISPOT assays of spleen and BM cells on days 4, 5 and 6 were performed. Number of Qβ-specific PCs (**C**) and spot diameter (**D**) after challenge with Qβ-RNA (black circles) or Qβ-PGA (open circles). Mean with SEM. P values were obtained using an unpaired t test. ** $p < 0.01$, *** $p < 0.001$. n = 4 mice per group. Data representative of 2 independent experiments.

Figure S2

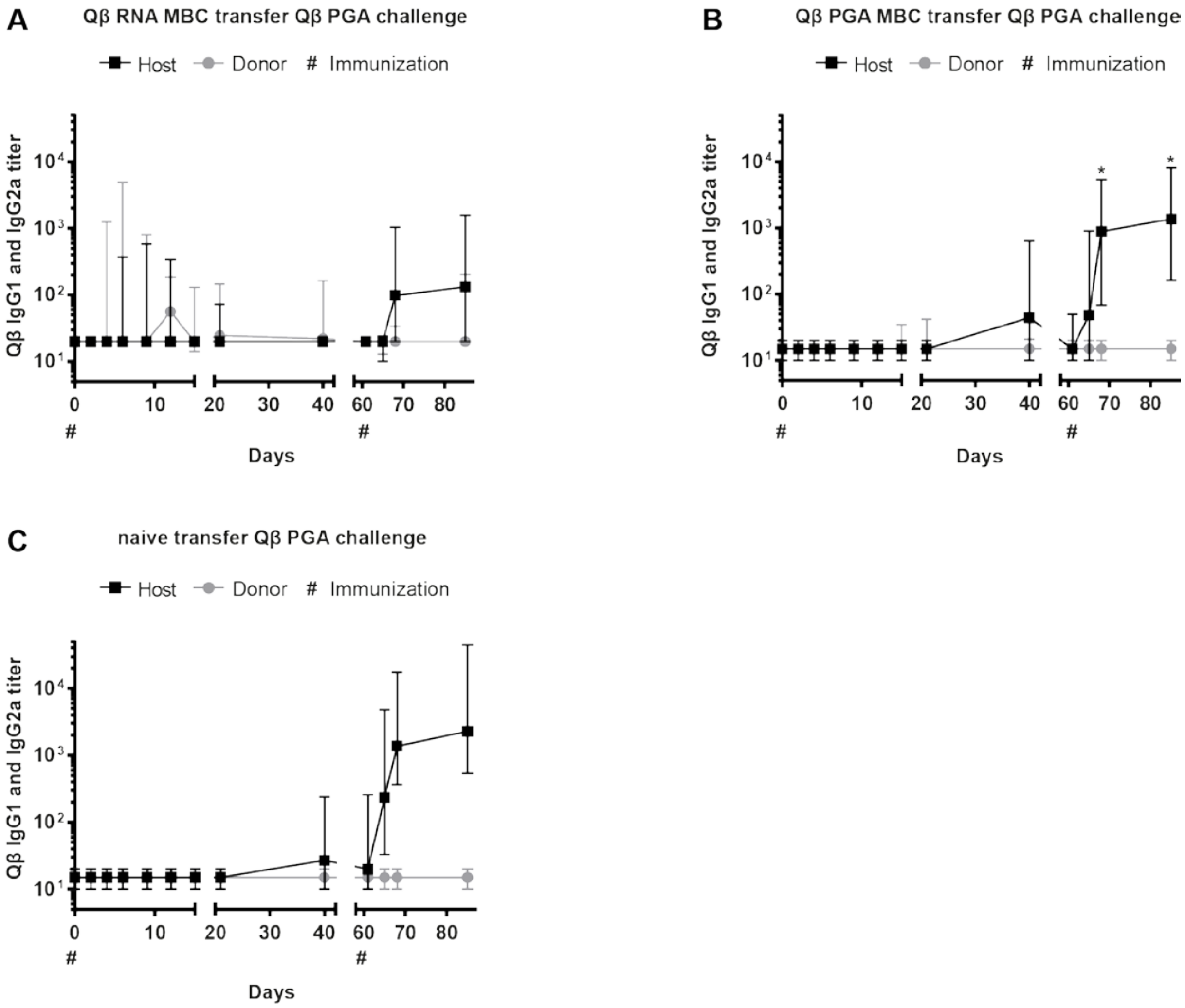

Figure S2. PC generation by immunization with Qβ-PGA. Memory B cells generated in presence (**A**) or absence (**B**) of RNA inside the VLP or naïve B cells (**C**) from IgHa donor mice were adoptively transferred into IgHb hosts. Recipient mice were challenged with 50 µg Qβ-PGA one day and 61 days after the transfer. (**A-C**) Anti-Qβ IgG1 and IgG2a titers were determined in the sera by ELISA. Allotype specific Ha and Hb detection antibodies were used to discriminate between donor and host derived antibodies. Mean with SEM. P values were obtained using an unpaired t test. * $p < 0.05$. n = 4 mice per group. Data representative of 2 independent experiments.
